# Pelvic Inflammatory Disease and Involuntary Infertility: Prospective Pilot Observations

**DOI:** 10.1155/S1064744995000494

**Published:** 1995

**Authors:** David Soper, Roberta B. Ness

**Affiliations:** 1 Department of Obstetrics and Gynecology Medical College of Virginia P.O. Box 980034 Richmond VA 23298-0034 USA; 2 Department of Epidemiology Graduate School of Public Health University of Pittsburgh Pittsburgh PA USA

## Abstract

*Objective:* We prospectively evaluated the rate of adverse reproductive outcomes following pelvic inflammatory disease (PID) in a small cohort of American women.

*Methods:* We enrolled 28 patients having either salpingitis confirmed by laparoscopy or endometritis confirmed by endometrial biopsy. The follow-up was maintained by clinic visits and telephone contact.

*Results:* A median of 15.4 months of follow-up was accomplished for 82.1% of these women. Fifty-two percent (13/25) had unprotected sexual activity without conception for at least 6 months. Fully 55.6% (10/18) of the cohort were involuntarily infertile after at least 1 year of follow-up.

*Conclusion:* In the first prospective cohort study of the reproductive outcomes of American women having had PID, high rates of infertility at 1 year of follow-up were experienced by these women.


Infectious Diseases in Obstetrics and Gynecology 3:145–148 (1995)
© 1995 Wiley-Liss, Inc.

### Pelvic Inflammatory Disease and Involuntary Infertility: Prospective Pilot Observations

**David Soper and Roberta B. Ness**

*Department of Obstetrics and Gynecology, Medical College of Virginia, Richmond, VA (D.S.), and Department of Epidemiology, Graduate School of Public Health, University of Pittsburgh, Pittsburgh, PA (R.B.N.)*

### Abstract

***Objective:*** **We prospectively evaluated the rate of adverse reproductive outcomes following pelvic inflammatory disease (PID) in a small cohort of American women.**

***Methods:*** **We enrolled 28 patients having either salpingitis confirmed by laparoscopy or endometritis confirmed by endometrial biopsy. The follow-up was maintained by clinic visits and telephone contact.**

***Results:*** **A median of 15.4 months of follow-up was accomplished for 82.1% of these women. Fifty-two percent (13/25) had unprotected sexual activity without conception for at least 6 months. Fully 55.6% (10/18) of the cohort were involuntarily infertile after at least 1 year of follow-up.**

***Conclusion:*** **In the first prospective cohort study of the reproductive outcomes of American women having had PID, high rates of infertility at 1 year of follow-up were experienced by these women.** © 1995 Wiley-Liss, Inc.

Key words

Salpingitis, endometritis, upper-genital-tract infection

Pelvic inflammatory disease (PID) affects almost 11% of American women of reproductive age[1] or an estimated 1 million women annually.[2,3] Tubal scarring or pelvic adhesions have been observed in one-third to one-half of women undergoing second-look laparoscopies after index episodes of PID.[4–6] The major reproductive sequelae resulting from this process of upper-genital-tract infection, inflammation, and obstruction include infertility, ectopic pregnancy, and chronic pelvic pain.

Only a limited number of observations are available on the frequency of adverse reproductive outcomes after episodes of treated PID. In the largest and longest prospective cohort study of PID ever conducted (Lund, Sweden: 1960–1984),[7] the rate of involuntary infertility was 12.6% after a mean follow-up of 7 years among 2,501 women. Of the women whose initial laparoscopic examinations revealed salpingitis, 16.0% experienced involuntary infertility. Of the women with no evidence of salpingitis at their baseline laparoscopies, 2.7% became involuntarily infertile. In a Canadian study,[8] 50 women with salpingitis were randomized to receive parenteral antibiotics and then followed for 5–7 months. Of the 68% of the women followed, 32.4% were involuntarily infertile. Finally, in the only United States study,[9] a retrospective cohort was constructed of 140 women admitted to San Francisco General Hospital for PID. The rate of infertility was estimated at 40%, but the reproductive outcomes were known only for the 36% of women who were successfully located.

This broad range of estimates of the prevalence of PID sequelae and the paucity of data from the United States represent an inadequacy in our knowledge of the epidemiology relating PID to infertil-

**Address correspondence/reprint requests to Dr. David E. Soper, Dept. of Obstetrics and Gynecology, Medical College of Virginia, P.O. Box 980034, Richmond, VA 23298-0034.**

Received June 6, 1995

Accepted September 20, 1995

ity. No prospective cohort studies have been conducted in the United States describing the natural history of treated PID. For these reasons, we conducted a longitudinal pilot study to characterize the reproductive outcomes of women with documented PID.

### SUBJECTS AND METHODS

The women were enrolled at the time they sought medical care at the Medical College of Virginia. To be included, a woman had to be nonpregnant and younger than 45 years, presenting with complaints of pelvic pain and gynecologic findings of uterine and adnexal tenderness.

The women were ascertained from the Emergency Department and enrolled in the prospective cohort if they had salpingitis by laparoscopy (11 patients) or endometritis by endometrial biopsy (17 patients). The diagnosis of salpingitis was based on Jacobson and Westrom's criteria,[10] which includes at least the following 3 signs during the baseline laparoscopy: 1) pronounced hyperemia of the tubal surface, 2) edema of the tubal wall, and 3) a sticky exudate on the tubal surface and from the fimbriated ends when patent. The severity of disease was graded according to the criteria of Westrom and Mardh.[11] The diagnosis of endometritis was based upon finding at least 1 plasma cell/×120 field and ≥5 neutrophils/×400 field in 6 stepped sections of endometrial tissue stained with hematoxylin and eosin.

The enrollment interview included demographic and historical information. The microbiologic assessment included an endocervical culture for *Neisseria gonorrhoeae* and *Chlamydia trachomatis*, as previously described.[6]

All subjects were treated as inpatients with a variety of antibiotics as part of ongoing randomized clinical trials. The women were then followed at least every 2 months by telephone or by clinic visit. The prospective interview data were collected regarding sexual activity, pregnancies, and contraceptive use.

The following procedures were instituted to maximize the proportion of women successfully followed. First, a single staff member of the study was responsible for maintaining contact with all women. Second, at the time of the baseline interview, the name and telephone number were elicited of 1 or preferably 2 friends or relatives who would be helpful in contacting the patient, if needed. Third, the patient, when necessary, was paid $10, for maintaining telephone contact. Fourth, the investigators made themselves available to help the patients obtain health care and to answer their medical questions.

**TABLE 1. Follow-up time, success, and mode in women with PID**

| | |
|---|---|
| Median follow-up time (months) | 13.4 |
| Overall follow-up success | 26/28 (92.9)[a] |
| Never contacted (post-72 h follow-up) | 2/28 (7.1) |
| Follow-up at least 6 months | 26/28 (92.9) |
| Follow-up at least 1 year | 18/28 (64.3) |
| Follow-up mode at last contact | |
| Telephone | 3/26 (11.5) |
| Clinic visit | 23/26 (88.5) |

[a]Number (%).

### Analysis

The primary outcome of interest was involuntary infertility, which was alternately defined as a failure to conceive after at least 6 months or a failure to conceive after 1 year of unprotected intercourse. The proportion of women who were infertile was calculated as a proportion of all women who contributed at least 6 months or at least 12 months to the respective analyses. Any woman who neither achieved pregnancy nor met the criteria for involuntary infertility was categorized as voluntarily infertile during a given follow-up period. The number of ectopic pregnancies that occurred during this longitudinal study were also recorded.

### RESULTS

### Demography

The patients ranged in age from 18 to 42 years. Over 90% of them were black.

### Follow-Up Success

Of the 28 women enrolled, 26 were interviewed within 8 weeks of the completion of the study, for an overall follow-up rate of 92.9% (Table 1). The two lost to follow-up were never located after leaving the hospital. The median follow-up time was 13.4 months (range 0–23.6 months). All of the 26 (92.9%) women successfully contacted after leaving the hospital were followed for at least 6 months, with 18 (64.3%) of them contributing a year of longitudinal data. The attrition at 1 year repre-

**TABLE 2. Reproductive outcomes among women with PID followed for at least 6 months and for at least 1 year**

| | | |
|---|---|---|
| Number of women sexually active for at least 6 months = 25 | | |
| Intrauterine pregnancy | = | 7/25 (28.0%) |
| Ectopic pregnancy | = | 1/25 (4.0%) |
| Involuntary infertility | = | 13/25 (52.0%) |
| Voluntary infertility | = | 4/25 (16%) |
| Number of women sexually active for at least 1 year = 18 | | |
| Intrauterine pregnancy | = | 7/18 (38.9%) |
| Ectopic pregnancy | = | 1/18 (5.6%) |
| Involuntary infertility | = | 10/18 (55.6%) |
| Voluntary infertility | = | 0 |

sented a mixture of lost to follow-up and a follow-up time falling short of 1 year. The predominant mode of last contact with cohort members was by clinic visit.

### Reproductive Outcomes

Intrauterine pregnancies occurred in 7 of 26 (26.9%) women for whom at least 6 months of longitudinal data were available (Table 2). One additional patient had an ectopic pregnancy. Of those who did not become pregnant, all but 1 were sexually active for at least 6 months. Thirteen women engaged in at least 6 months of sexual activity without contraception but did not conceive. Thus, 28% (7/25) of the sexually active cohort had had intrauterine pregnancies, 52.0% (13/25) had had no conception despite having had unprotected intercourse, and 20.0% (5/25) had had voluntary infertility for at least 6 months. For those contributing a year of follow-up experience, 10 remained nongravid despite being at risk for pregnancy, resulting in a rate of involuntary infertility of 55.6% (10/18) in the group contributing the longest prospective experience.

The strongest baseline correlate of subsequent infertility was a history of PID. Three of 12 (25%) women who became infertile recalled at least 1 previous episode of disease, whereas none of the women who became pregnant did so. The cervical cultures yielding *N. gonorrhoeae* were similarly common in women experiencing infertility (66.7%) and in those becoming pregnant (57.1%). In addition, the cervical cultures for *C. trachomatis* were similarly common in women experiencing infertility (25.0%) and intrauterine pregnancy (28.6%). The patient who developed an ectopic pregnancy had a positive culture for *N. gonorrhoeae*, a history of PID, and a grade-3 (severe) salpingitis.

### DISCUSSION

This first American prospective cohort study of reproductive sequelae after PID demonstrates 2 important points. First, it shows that, in women with documented salpingitis, the rates of involuntary infertility over 6 months and 1 year were quite high. Second, it demonstrates substantial success in following a small group of young, urban, minority women. The strategies employed in the prospective evaluation of these women provide a guide for future longitudinal studies of women with sexually transmitted diseases.

The infertility rates in our study were substantially higher than those reported in the only other available prospective cohort study with at least 1 year of longitudinal information. That study from Sweden[8] revealed an infertility rate of 16% after a mean of 7 years of follow-up. Closer to our findings are those of the studies from Canada[8] and the United States[9] of women hospitalized for salpingitis or PID suggesting infertility rates of 34% and 40%, respectively, after 5 months to 2 years of unprotected intercourse. There are several possible reasons for these disparate findings. First, the infertility rate defined as no conception for 1 year will be higher than the rate defined as no conception for a longer period (as was the case in Sweden) because the infertility rate would decrease with the longer time spent attempting pregnancy.[12,13] However, because 1 year of involuntary infertility is frequently used as a benchmark for the initiation of reproductive diagnostics and therapeutics in the United States, the infertility rates at 1 year may have greater meaning from the perspective of health services than infertility rates after many years would have. Second, the patients hospitalized for clinical reasons may have been more ill, thus experiencing higher rates of infertility, than the patients presenting to institutions that hospitalize all women as a standard practice, as was the case in Sweden. Third, the women in our study may have experienced more episodes of PID previous to their study entry than the women in studies reporting lower rates of infertility. This consideration is difficult to assess because PID frequently goes unrecognized and the recalled history of disease provides a substantial underestimate of frequency.[14] Finally, our patients may have had a different spectrum of microbiologic pathogens than the women enrolled in Sweden. However, this explanation is doubtful because a higher proportion of the women in our study had gonorrhea-associated disease which is less likely to result in infertility[8,15,16] than the chlamydia-associated disease that became prevalent in Sweden during the later years of that cohort investigation.[17] Whatever the reason, the very high rate of subsequent infertility among women with salpingitis in our study is of substantial concern.

Our high follow-up rates contradict a widespread belief that United States women with PID cannot be followed on a long-term basis with any reasonable success. The effective methods employed to follow this sample of patients are easily applicable and widely available to other researchers. We strongly urge further studies of PID and the reproductive morbidities such as infertility and ectopic pregnancy that we, as clinicians and public-health providers, are trying to prevent.

### REFERENCES

1. Aral SO, Mosher WE, Cates W: Self-reported pelvic inflammatory disease in the US: A common occurrence. JAMA 266:2570–2573, 1991.
2. Sweet RL: Pelvic inflammatory disease and infertility in women. Infect Dis Clin N Am 1:199, 1987.
3. Blount JH, Reynolds GH, Rice RJ: Pelvic inflammatory disease: Incidence and trends in private practice. MMWR 32(4SS):27SS, 1986.
4. Wolner-Hanssnen P, Westrom L: Second look laparoscopy after acute salpingitis. Obstet Gynecol 61:702–704, 1983.
5. Brihmer C, Brundin J: Second look laparoscopy after treatment of acute salpingitis with doxycycline/benzylpenicillin procaine or trimethoprim-sulfamethoxazole. Scand J Infect Dis 53:65–69, 1988.
6. Soper D, Brockwell NJ, Dalton HP: Microbial etiology of urban emergency department acute salpingitis: Treatment with ofloxacin. Am J Obstet Gynecol 167:653–660, 1992.
7. Westrom L, Joesoef R, Reynolds G, Hagdu A, Thompson SE: Pelvic inflammatory disease and fertility. A cohort study of 1,844 women with laparoscopically verified disease and 657 control women with normal laparoscopic findings. Sex Trans Dis 19:185, 1992.
8. Brunham RC, Binns B, Guijon F, Danforth D: Etiology and outcome of acute pelvic inflammatory disease. J Infect Dis 158:510, 1988.
9. Safrin S, Schachter J, Dahrouge D, Sweet RL: Long-term sequelae of acute pelvic inflammatory disease: A retrospective cohort study. Am J Obstet Gynecol 166: 1300, 1992.
10. Jacobson L, Westrom L: Objectivized diagnosis of acute pelvic inflammatory disease. Am J Obstet Gynecol 105: 1088, 1969.
11. Westrom L, Mardh PA: Salpingitis. In Holmes KK, Mardh P-A, Sparling PF, et al. (eds): Sexually Transmitted Diseases. 1st ed. New York: McGraw-Hill, p 615, 1984.
12. Barad DH: Epidemiology of infertility. Infert Reprod Med Clin N Am 2:255, 1991.
13. Cates W Jr, Rolfs RT Jr, Aral SO: Sexually transmitted diseases, pelvic inflammatory disease, and infertility: An epidemiologic update. Epidemiol Rev 12:199, 1990.
14. Wolner-Hanssen P, Kiviat NB, Holmes KK: Atypical pelvic inflammatory disease: Subacute, chronic, or subclinical upper genital tract infection in women. In Holmes KK, Mardh PA, Sparling PF, Wiesner PJ, Cates W, Lemon S, Stamm W (eds): Sexually Transmitted Diseases. 2nd ed. New York: McGraw-Hill, p 615, 1989.
15. Cates W Jr: Sexually transmitted organisms and infertility: The proof of the pudding. Sex Trans Dis 11:113–116, 1984.
16. Svensson L, Mardh P-A, Westrom L: Infertility after acute salpingitis with special reference to *Chlamydia trachomatis*. Fertil Steril 40:322, 1985.
17. Westrom L: Incidence, prevalence, and trends of acute pelvic inflammatory disease and its consequences in industrialized countries. Am J Obstet Gynecol 138:880, 1980.
